# The Rapidly Evolving Landscape of DCD Heart Transplantation

**DOI:** 10.1007/s11886-024-02148-w

**Published:** 2024-10-09

**Authors:** Yashutosh Joshi, Katherine Wang, Campbell MacLean, Jeanette Villanueva, Ling Gao, Alasdair Watson, Arjun Iyer, Mark Connellan, Emily Granger, Paul Jansz, Peter Macdonald

**Affiliations:** 1 Heart Transplantation Unit, St Vincent’s Hospital Sydney, 390 Victoria St., Darlinghurst, NSW 2010 Australia; 2 Victor Chang Cardiac Research Institute, Darlinghurst, NSW Australia; 3 University of New South Wales, Randwick, NSW Australia

**Keywords:** Donation after circulatory death, Heart transplantation, Normothermic machine perfusion, Hypothermic machine perfusion, Normothermic regional perfusion

## Abstract

**Purpose of Review:**

To summarise current international clinical outcomes from donation after circulatory death heart transplantation (DCD-HT); discuss procurement strategies, their impact on outcomes and overall organ procurement; and identify novel approaches and future areas for research in DCD-HT.

**Recent Findings:**

Globally, DCD-HT survival outcomes (regardless of procurement strategy) are comparable to heart transplantation from brain dead donors (BDD). Experience with normothermic machine perfusion sees improvement in rates of primary graft dysfunction. Techniques have evolved to reduce cold ischaemic exposure to directly procured DCD hearts, though controlled periods of cold ischaemia can likely be tolerated. There is interest in hypothermic machine perfusion (HMP) for directly procured DCD hearts, with promising early results.

**Summary:**

Survival outcomes are firmly established to be equivalent between BDD and DCD-HT. Procurement strategy (direct procurement vs. regional perfusion) remains a source of debate. Methods to improve allograft warm ischaemic tolerance are of interest and will be key to the uptake of HMP for directly procured DCD hearts.

## Introduction

Over the last decade, there has been growing global uptake of heart transplantation from donation after circulatory death donors in an effort to address the increasing demand for donor hearts [1–4].

The last review from our unit on DCD heart transplantation focussed on the long journey of DCD hearts [5]. The world’s first heart transplant performed by Christiaan Barnard [6] was in fact from a DCD donor. However, concerns surrounding the impact of warm ischaemia on cardiac allografts, and subsequent clarification of the definition of brain death [7], led to the use of brain-dead donors (BDD) and static cold storage (SCS) for heart transplantation in the decades that followed.

Pre-clinical, large animal work conducted by our laboratory allowed for the clinical translation of normothermic machine perfusion (NMP) as a means to assess the viability of hearts procured via a DCD pathway after being subjected to inevitable periods of warm ischaemia [8–10]. This resulted in the world’s first DCD heart transplantation program utilising the Transmedics Organ Care System Heart (OCS Heart) (Boston, MA, USA) NMP device in Sydney, which was subsequently adopted by the Papworth group in the United Kingdom [10, 11].

Positive early outcomes have resulted in DCD donors becoming firmly integrated into the current global landscape of heart transplantation. With hypothermic machine perfusion (HMP) entering the field for BDD heart transplantation [12, 13], there is evolving interest in exploring, and comparing, alternative preservation modalities for DCD donors (beyond NMP). Additionally, procurement strategies remain a source of contention, and ethical debate, with potential impact on the retrieval of lungs and abdominal organs.

In this review we shall explore the latest clinical experience and highlight research and progress that has been made since our previous review, with a particular focus on clinical outcomes and the efforts being made in determining the ideal procurement and preservation approaches for DCD hearts.

## Clinical Outcomes

It is evident that DCD heart transplantation has successfully expanded the donor pool, increasing heart transplantation activity globally [1, 14, 15]. In the United States (US), United Network for Organ Sharing (UNOS) Registry data analysis demonstrates that DCD heart transplantation has significantly contributed to a reduction in wait-list time, particularly in candidates who would typically have longer expected wait-list times; these are candidates who are wait-list status 4 (stable with a VAD) and greater [16, 17]. In our experience, since 2021, the majority of heart transplants performed on patients who would qualify as status 1 (critically ill on mechanical support), were from DCD donors [18].

Described in our previous review [5], two commonly used methods of DCD heart procurement include: (I) DPP-NMP: Direct procurement pathway (DPP) followed by NMP and (II) taNRP-CS: Thoracoabdominal normothermic regional perfusion followed by cold storage (CS), most commonly referring to static cold storage (SCS).

### Direct Procurement Protocol Followed by Normothermic Machine Perfusion

Our unit in Australia has the longest experience with DCD heart transplantation, with our program now having over a decade of experience. DCD hearts are procured using a DPP followed by NMP. Our published data reporting on DCD heart transplants from 2014 to 2022 demonstrates that there has been no significant difference in the 8-year survival outcomes of DCD heart transplant recipients compared to BDD receipients [1]. Since 2021, our unit has had access to HMP for BDD with a predicted prolonged donor ischaemic time (DIT). In comparing our heart transplantation outcomes from recipients of DCD hearts procured using NMP, BDD hearts preserved with either HMP or SCS, there appears to be no significant difference in survival during this new modern era of machine perfusion [18]. 

In the initial Sydney experience reported by Chew et al. [19], there was a high rate (35%) of severe primary graft dysfunction (sPGD) requiring mechanical circulatory support (MCS) - this is often cited in the literature as a concern with DCD heart transplantation. However, as our experience and familiarity with NMP and DCD donation grew, there was a statistically significant improvement in sPGD [1]. When directly compared to the initial cohort, a more contemporary cohort had a significantly reduced rate of sPGD at 8%; this improvement has continued and since 2021, our DCD program now has a 7% rate of sPGD [1, 18].

The ongoing reduction in Australian DCD heart transplant sPGD rates can be attributed to the efforts being made by all parties involved in the organ retrieval process and reflects growing experience. Particular attention has been paid to reducing the warm ischaemic time. An asystolic warm ischaemic time (aWIT) of > 15 min has been consistently identified as a vital, and statistically significant predictor of sPGD [1, 18, 19]. Where possible, and when local policy permits, there is an increased effort being made to advocate for withdrawal of life support (WLS) to occur in the anaesthetic bay and on an operating table as our experience has shown withdrawal location to play a significant role in influencing aWIT [20]. Retrieval techniques have also been refined in order to minimise aWIT. Furthermore, where once it was a novel phenomenon, our exposure to NMP over the last decade has resulted in a greater understanding of the nuances in the ex-situ management of the DCD heart, as experience accumulated with each retrieval and subsequent transplant, including any organ rejections following NMP assessment.

The Papworth group in the United Kingdom (UK) have also reported there to be no significant difference in survival when comparing recipients of BDD to DCD donor hearts [15]. In 2023, results from an 18 month observational national pilot study in the UK were published, this reported on outcomes after the DCD heart retrieval service was nationalised (as opposed to DCD heart transplants occurring at a single centre) [2]. DCD hearts were procured utilising DPP-NMP with results from this multi-centre study once again demonstrating there to be no significant difference in survival between recipients of BDD and DCD donor hearts [2]. Furthermore, there were no survival differences when compared to pre-pilot era outcomes [2].

Though it did not seem to influence survival, there was a significantly increased rate of extracorporeal membrane oxygenation (ECMO) in recipients of DCD hearts during the pilot study (40%) [2]. This was thought to be likely secondary to higher ECMO rates in centres that were not experienced with DCD heart transplantation [2]. The results and the authors highlight the importance of experience, and advocate for centres that are new to DCD heart transplantation to learn from established DCD heart transplant units [2]. Encouragingly, this study demonstrates that with appropriate training and expertise in DCD retrievals, the national expansion of DCD programs is feasible without compromising survival outcomes, resulting in improved access to an expanding donor pool.

Importantly, 2023 also marked the publication of the first randomised control trial (RCT) involving DCD heart transplantation, further validating DCD heart transplantation as a standard of care for heart transplant units [4]. This was a multi-centre trial based in the United States [4]. Patients assigned to the DCD heart transplantation group received a DCD heart procured via DPP followed by NMP using the OCS Heart and compared to recipients of BDD retrieved hearts following SCS; DCD heart transplantation was found to be non-inferior when considering the primary end-point of 6 month survival [4]. Overall, the sPGD rate amongst DCD heart transplant recipients in the trial [4] was 15%, compared to 5% in the BDD group – this is likely a reflection of the early experience with DCD retrievals and NMP (which has been observed in early outcomes internationally [2, 19]), as well as the multi-centre nature of the trial. Whilst it does not seem to have impacted survival, it is likely that the rate of sPGD will continue to improve with broadening use of DCD donors.

### Thoracoabdominal Normothermic Regional Perfusion Followed by Cold Storage

Considering taNRP specifically, results from an international multicentre retrospective observational study of 157 DCD taNRP-CS hearts conducted across the United States, Belgium, Spain and the UK have been published [14]. Across the 15 centres enrolled in this study, there was a 23% increase in heart transplantation activity with no differences in survival up to 5yrs between recipients of DCD and BDD heart transplants [14].

The rapid uptake of DCD heart transplantation has resulted in a large amount of registry data from the United States now being available for analysis [21, 22]. A recent analysis of the United States Organ Procurement and Transplantation Network (OPTN) heart transplantation data between December 2019-September 2023 demonstrated there to be no significant difference in 3 year survival or rates of primary graft dysfunction (PGD) between recipients of DCD and BDD hearts [22]. This was regardless of DCD procurement method with taNRP and DPP accounting for 249 and 543 DCD heart transplants respectively (compared to 10,833 transplants from BDD) [22].

A large single centre study from the Vanderbilt University Medical Centre serves as an example of a centre being able to facilitate both taNRP as well as DPP approaches for DCD retrievals [23]. Out of 122 DCD heart transplants performed in the study, taNRP-CS accounted for 101 (83%) with 21 (17%) of DCD transplants occurring via DPP-NMP. This was compared to transplant outcomes from BDD hearts procured using either NMP (10/263, 4%) or SCS (253/263, 96%) [23]. Consistent with recent international literature, there were no significant differences in 1 year survival between the two groups [23].

The international analysis of taNRP outcomes by Louca et al. [14] reported no significant differences in the incidence of post-transplant MCS between the DCD and BDD groups (12.8% and 12.7% respectively). The Vanderbilt group also reported no differences in sPGD rates between the two groups (6% across both) [23]. The Vanderbilt study however was not powered to report differences between DCD procurement techniques [23]. This is where rapidly growing registry data and analysis can help provide further answers with the most recent US OPTN analysis showing no significant difference in survival or PGD rates when comparing DCD DPP-NMP and DCD taNRP-CS outcomes [22].

### Cold Ischaemia in Directly Procured DCD Hearts Preserved with NMP

**Fig. 1:**
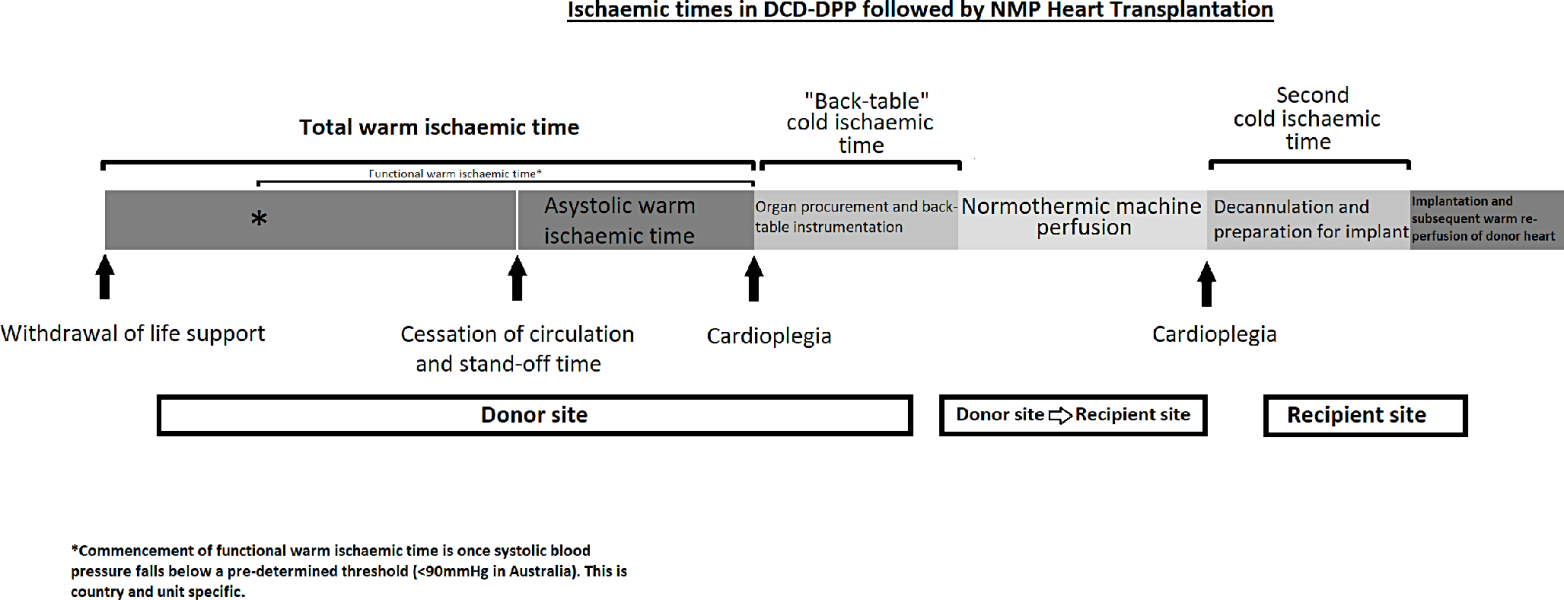
Retrieval timeline for directly procured donation after circulatory death donor hearts

In DCD Hearts procured via DPP, donor hearts are also subject to short cold ischaemic periods (see Fig. 1). The first exposure is following the administration of cardioplegia during the initial organ procurement. Following this the heart is then placed in a cold slurry and instrumented prior to reperfusion on the OCS Heart NMP device. In our experience this can be referred to as the back table cold ischaemic time (CIT). Following this, the heart is then re-perfused and assessment on the NMP device ensues. The donor heart is then subject to a second cold ischaemic period as cardioplegia is once again administered in order to facilitate decannulation from the OCS Heart once the implanting surgeon is ready. In our experience, prior to this second administration of cardioplegia, the OCS Heart is connected to a water-heater cooler and the heart is cooled to 16 degrees Celsius [1]. During the implant, cold blood cardioplegia is then administered at regular intervals and a “hot shot” dose of dilute warm blood cardioplegia is then administered prior to the removal of the cross clamp at the discretion of the surgeon.

Exposure of the DCD heart to two cold ischaemic times results in two potential subsequent periods of ischaemia reperfusion injury (IRI). Of late, questions have arisen as to whether this IRI exposure, along with the duration of CIT, may potentially contribute to primary graft dysfunction [2, 24]. This issue is discussed in the UK national pilot study where the time to reperfusion on the OCS Heart following procurement was influenced by the method retrieval teams would use to mount the cardiac allograft onto the NMP device [2]. “Method A” (previously described by Messer et al. [15]) involved the inferior and superior vena cavae (IVC and SVC) being left open, along with the pulmonary artery (PA) cannula being disconnected; this allowed the right ventricle (RV) to remain in an unloaded state. In “Method B” (similar to the approach in Sydney [1] and other centres [4, 25]), the cavae are oversewn/ligated and the PA cannula attached to a return connector on the OCS Heart allowing for coronary flow to be measured.

There were no significant differences in survival or sPGD outcomes reported between the two methods in the pilot study [2], however Method B was found to have a significantly longer time from a systolic blood pressure (SBP) < 50mmHg up to reperfusion on the OCS Heart (which was defined in this particular study as the functional warm ischaemic time [fWIT]). This significant increase in time (median of 25 min for Method A vs. 33 min for Method B) is due to the extra steps taken to instrument the allograft for the OCS Heart. Messer et al. [2] suggested that a non-significant trend for lower 30 day survival with Method B may reach significance with longer follow up, however, longer term data from the Sydney experience suggests that this is not the case [1]. In our experience, increasing CIT and increasing CIT + fWIT was not associated with an increased risk of sPGD (fWIT defined in our unit as time from SBP < 90mmHg to administration of cardioplegia) [1, 18]. Furthermore, our more contemporary cohort, which has experienced a significantly lower rate of sPGD, had a significantly higher CIT exposure compared to our initial cohort [1]. 

The heart transplant team at Stanford University (Stanford, CA, USA) described an innovative operative approach to avoid a second period of cold ischaemia [24, 26]. Krishnan et al. [26] describe an original set up that allows for concurrent cardiopulmonary bypass (CPB) to be initiated in the recipient, alongside ongoing normothermic perfusion on the OCS Heart via a shared CPB circuit. Briefly, upon return to the recipient operating theatre, an aortic root needle is introduced to the donor heart and a cross clamp applied - this allows for antegrade perfusion of the donor heart from the CPB circuit [26]. The donor heart is then removed from the OCS device and implanted with ongoing antegrade perfusion [26]. Once the left atrial and aortic anastomoses are complete, donor heart and recipient cross clamps are removed and the heart is re-perfused via the aortic cannula while the remainder of the anastomoses are completed [26]. This novel approach describes the first instance of a “beating-heart” transplant and results in the DCD donor heart being exposed to just one period of CIT (at the donor hospital site). Outcomes appear to be promising with a case series of 10 DCD heart transplants utilising this method being described with no instance of primary graft dysfunction or early mortality [24].

The impact of CIT in DCD hearts preserved with NMP is an area for future potential research. Whilst Krishnan et al. [24] suggest that the second period of CIT may potentially contribute to graft dysfunction, data from our contemporary experiences with DCD heart transplantation demonstrate that exposure to CIT does not adversely impact outcomes, with excellent survival and sPGD rates despite two periods of CIT [1, 18]. We believe this suggests: the DCD heart is likely able to tolerate periods of controlled cold ischaemia; that ischaemic preconditioning may confer some cardioprotection following a second CIT [27]; and, that the asystolic warm ischaemic time likely plays a more important role in predicting sPGD [1, 18]. Novel techniques in “beating-heart” transplantation that limit a second CIT however, do appear to be feasible and safe [24, 26].

### Ethics of taNRP and Impact on Concurrent Retrieval of Other Organs

The ethics surrounding taNRP remains a contentious topic, with concerns and discussion focussing on whether the in-situ re-animation of donor hearts – and the potential for any blood flow (including through collateral vessels) to the cerebral circulation – violates the “dead donor rule.” [28–31].

One potential argument supporting the use of taNRP is the increased utilisation of donor hearts compared to a DPP approach. Bakhtiyar et al. [22] reported a 1.64% rate of non-use in the DCD taNRP group compared to 10.83% in the DCD-DPP group. Arguments favouring normothermic regional perfusion (NRP) suggest that it is a technique that maximises organ utilisation of the heart as well as abdominal organs [32]. DCD livers procured through NRP are known to have significantly reduced rates of 30-day graft loss and significantly lower rates of ischaemic cholangiopathy [33]. Furthermore, UNOS registry data shows, compared to DPP-NMP procurement, when taNRP is used for the retrieval of DCD hearts, there is a significantly increased utilisation rate of concurrently procured DCD livers, as well as a reduced rate of delayed graft function in DCD kidneys [34].

Whilst there appears to be a benefit to abdominal organs via, the impact of taNRP on the retrieval of DCD lungs is less well known. This is due to concerns surrounding the potential risk of pulmonary oedema from fluid used to prime CPB or ECMO circuits. Cain et al. [35] describes a small case series from the University of Colorado Denver where lung transplants were performed successfully following a taNRP procurement technique with a specific focus on reducing pulmonary oedema. Strategies utilised in the Colorado case series included: early and aggressive donor diuresis pre-procurement; early venous cannulation and donor decongestion; early venting of the donor PA, and, early diuresis post-transplant [35]. Overall however, UNOS data suggests concurrent DCD lung utilisation rates are low regardless of procurement technique (14.9% and 13.8% for taNRP and DPP approaches respectively) [36]. With no significant differences in 6-month survival in taNRP retrieved lung transplants compared to DPP [36], procurement rates from both techniques have the potential to improve. Emerging interest in strategies to minimise pulmonary oedema during taNRP is promising and highlights the need to consider the lungs (and not just the heart and abdominal organs) in future decisions and ethical discussions surrounding which retrieval technique maximises organ procurement.

From an alternative viewpoint, arguments can be made that taNRP and the subsequent implications of in-situ re-perfusion of organs, challenge the definition, as well as our understanding of death [37, 38]. This is a discussion that will likely continue. For the time being, national and state laws currently govern the permissibility of taNRP; in Australia for example, taNRP is not legally allowed. Studies in centres where taNRP is permitted provide further understanding and may help guide future policy. A Spanish study by Royo-Villanova et al. [39] describes one of the first studies to invasively measure intracranial blood pressure (ICBP) during 10 clinical DCD normothermic regional perfusion cases, during this time no change in ICBP was recorded, providing evidence that cerebral blood flow was not restored. Frontera et al. [40] similarly showed there to be no flow on intraoperative transcranial doppler in two clinical DCD taNRP cases. This has set the stage for future intraoperative studies which will likely provide further insight [41]. 

### Hypothermic Machine Perfusion and DCD Heart Transplantation

Hypothermic machine perfusion has emerged as a promising donor heart preservation strategy in hearts procured from BDD. The XVIVO Heart Assist Transport device (XHAT, Gothenburg, Sweden) provides temperature-controlled preservation at ~ 8^o^C and is currently the only commercially available preservation device to also provide ongoing hypothermic machine perfusion, providing a constant infusion of a hyper-oncotic, oxygenated, blood-based solution to the coronary circulation throughout the preservation time [12, 13, 42].

Results from a recently completed trial in Australia and New Zealand is promising for showing HMP to have a role in the setting of extended donor preservation times [13]. Our unit in Sydney specifically has shown positive results with a mean donor preservation time of over 6 h and a 5% rate of sPGD [18]. Furthermore, a European RCT demonstrated that the use of HMP in BDD heart transplantation reduces rates of PGD compared to SCS [43].

Naturally, this raises the question as to whether there is a role for HMP in DCD heart transplantation. The use of HMP, as opposed to SCS, following a DCD-taNRP procurement appears to be a potentially feasible solution for retrievals where a prolonged donor heart preservation time is expected. With the heart having already being assessed in-situ, the question regarding organ viability following the DCD withdrawal process is already answered prior to any further hypothermic preservation strategy. However, it is when HMP for DCD hearts following a direct procurement is considered, that the role is less clear.

Following Christiaan Barnard’s first heart transplant from a DCD donor, a major limiting factor in the wide-spread utilisation of DCD donor hearts up until 2014 was due to: (I) concerns regarding the viability of cardiac allografts following a global, warm ischaemic insult, and (II) not knowing the acceptable time limits for this period of warm ischaemia. An advantage of NMP is the ability to assess a donor heart prior to transplantation. Described in our previously published experience [1, 19], in Sydney, this is done through: visually assessing RV contractility, as well as an assessment of haemodynamic parameters and lactate profiles. Once the heart is procured, this assessment through NMP is possible regardless of the warm ischaemic time endured, and with a clinical understanding of an increased risk of sPGD with an aWIT of > 15 min, decisions can be made based on warm ischaemia times as well as the heart’s performance on the OCS Heart NMP device.

An assessment of organ viability is currently not possible during HMP. However, despite this, large animal studies in Denmark have shown DCD-DPP transplantation to be possible [44]. A porcine transplant series by Moeslund et al. [44] demonstrated that compared to NRP followed by SCS, recipient pigs receiving a DCD heart preserved with HMP following either DPP or NRP, had significantly improved post-transplant biventricular function. The median fWIT (defined in the study as SBP < 50mmHg to administration of cardioplegia) in the DPP-HMP group was 19 min, the aWIT time was set at 15 min [44].

The first clinical DCD-DPP heart transplant utilising HMP was performed in Leuven, Belgium [45]. Informed by experiences from international DCD programs, the maximum fWIT was set at 30 min (defined as SBP < 50mmHg to administration of cardioplegia) and a donor upper age limit of 55yrs; antemortem interventions such as coronary angiography and administration of antemortem heparin were permissible [45]. The Leuven group reported a case series of 3 successful DCD heart transplants following HMP, short term results were excellent with no incidence of PGD and 100% survival at 30-days [45].

The rapid adoption of HMP into clinical practice for DCD-DPP heart transplants show promising results. The ability to perform antemortem interventions will be important for DCD-DPP HMP heart transplantation. In the Sydney experience, older DCD donors are increasingly being utilized and ex-situ coronary angiography is a useful adjunct that allows this to occur [46, 47]. Currently this has not been shown to be possible with HMP and thus pre-retrieval assessment, thorough investigation and careful selection is vital as DCD-DPP HMP gathers more experience.

In the DCD-DPP HMP clinical series, the maximum fWIT was 21 min, with a maximum aWIT of 14 min [45]. This is an area of interest as in the DCD porcine transplant experiments, Moeslund et al. [44] noted two donor animals in the DPP-HMP group with a fWIT > 25 min that could not be weaned off CPB due to ischaemic contractures. Clinically, this would have been a heart that could have been assessed with NMP prior to reaching a decision regarding transplantation. In our experience to date, DCD hearts have been successfully transplanted with an aWIT of up to 20 min (without incidence of sPGD); whilst our data suggests an aWIT of > 15 min increases the risk of sPGD, assessment on NMP allows the transplant team to decide following consideration of all factors (ischaemic timings, recipient urgency and performance on OCS heart).

Currently for DCD-DPP HMP, once a heart is procured, barring any concerns with perfusion on the XHAT device, transplantation will inevitably follow without an assessment of the donor organ. Therefore, in the ongoing progression of DCD-DPP HMP, particular attention will need to be paid to investigating the acceptable limits of warm ischaemia for this preservation modality. If there is a limit, consideration of potential pharmacological supplementation, potential novel biomarkers to assess viability, or resuscitative strategies, can aim in ensuring that all DCD hearts can be procured.

Whilst XHAT allows for HMP, another form of hypothermic preservation currently being used is temperature-controlled storage (TCS). The SherpaPak by Paragonix (MA, USA) is an example of this, preserving the donor heart at a consistent temperature of ~ 5^o^C whilst the heart is submerged in a preservation solution (without perfusion) [48]. Yet to be utilised in a clinical DCD setting, data is promising in clinical BD heart transplants – demonstrating significantly lower rates of sPGD compared to SCS when being utilised for marginal BD donors heart transplants despite significantly higher preservation times [49]. However with no way to determine the viability of DCD hearts, much like XHAT with HMP, further research will be needed to determine the role of TCS in DCD heart transplantation.

## Conclusions

DCD heart transplantation has seen a rapid uptake in the last decade with a sharp increase in the availability of clinical data over the last 5 years. Clinical outcomes remain excellent with survival outcomes consistently and globally shown to be similar to heart transplants from BD donors and a significant increase in transplantation activity as a result of the expansion of the donor pool. Heart transplantation from DCD donors should be considered as standard practice for busy heart transplant units. Future developments will focus on elucidating long-term survival outcomes and refining optimal procurement and preservation strategies, offering opportunities for ongoing improvement and innovation.

## Key References

Joshi Y, Scheuer S, Chew H, et al. Heart Transplantation From DCD Donors in Australia: Lessons Learned From the First 74 Cases. Transplantation 2022:10.1097/TP.0000000000004294. DOI: 10.1097/tp.0000000000004294.

**Describes DCD heart transplantation outcomes from Australia**,** comparing results from an earlier published series against a contemporary group. Describes technical aspects of normothermic machine perfusion and assessment. Establishes asystolic warm ischaemic time as an important predictor of primary graft dysfunction.**

Messer S, Rushton S, Simmonds L, et al. A national pilot of donation after circulatory death (DCD) heart transplantation within the United Kingdom. J Heart Lung Transplant 2023;42(8):1120–1130. (In eng). DOI: 10.1016/j.healun.2023.03.006.

**Reports pilot experience outcomes from a national DCD heart transplantation program in the United Kingdom.**

Schroder JN, Patel CB, DeVore AD, et al. Transplantation Outcomes with Donor Hearts after Circulatory Death. N Engl J Med 2023;388(23):2121–2131. (In eng). DOI: 10.1056/NEJMoa2212438.

**First randomised control trial of DCD vs. BD Donor heart transplantation.**

Louca J, Öchsner M, Shah A, et al. The international experience of in-situ recovery of the DCD heart: a multicentre retrospective observational study. EClinicalMedicine 2023;58:101887. (In eng). DOI: 10.1016/j.eclinm.2023.101887.

**Describes clinical outcomes from an international experience with taNRP-DCD heart transplantation.**

Brouckaert J, Vandendriessche K, Degezelle K, et al. Successful clinical transplantation of hearts donated after circulatory death using direct procurement followed by hypothermic oxygenated perfusion: A report of the first 3 cases. The Journal of Heart and Lung Transplantation 2024. DOI: 10.1016/j.healun.2024.07.018.

**Reports the first clinical series of directly procured DCD heart transplants utilising HMP**.

## Data Availability

No datasets were generated or analysed during the current study.

## References

[1] Joshi Y, Scheuer S, Chew H, Ru Qiu M, Soto C, Villanueva J, et al. Heart Transplantation from DCD donors in Australia: lessons learned from the first 74 cases. Transplantation. 2022. 10.1097/TP.0000000000004294. 36044329 10.1097/TP.0000000000004294

[2] Messer S, Rushton S, Simmonds L, Macklam D, Husain M, Jothidasan A, et al. A national pilot of donation after circulatory death (DCD) heart transplantation within the United Kingdom. J Heart Lung Transpl. 2023;42(8):1120–30. 10.1016/j.healun.2023.03.006. 10.1016/j.healun.2023.03.006 37032222

[3] Miñambres E, Royo-Villanova M, Pérez-Redondo M, Coll E, Villar-García S, Canovas SJ, et al. Spanish experience with heart transplants from controlled donation after the circulatory determination of death using thoraco-abdominal normothermic regional perfusion and cold storage. Am J Transpl. 2021;21(4):1597–602. 10.1111/ajt.16446. 10.1111/ajt.16446 33319435

[4] Schroder JN, Patel CB, DeVore AD, Bryner BS, Casalinova S, Shah A, et al. Transplantation outcomes with Donor hearts after Circulatory Death. N Engl J Med. 2023;388(23):2121–31. 10.1056/NEJMoa2212438. 37285526 10.1056/NEJMoa2212438

[5] Joshi Y, Villanueva J, Gao L, Hwang B, Zhao C, Doyle A, et al. Donation after circulatory death: a New Frontier. Curr Cardiol Rep. 2022. 10.1007/s11886-022-01798-y. 36272050 10.1007/s11886-022-01798-y PMC9747832

[6] Barnard CN. The operation. A human cardiac transplant: an interim report of a successful operation performed at Groote Schuur Hospital, Cape Town. S Afr Med J. 1967;41(48):1271–4. 4170370

[7] A definition of irreversible coma. Report of the Ad Hoc Committee of the Harvard Medical School to examine the definition of Brain Death. JAMA. 1968;205(6):337–40. 5694976

[8] Iyer A, Gao L, Doyle A, Rao P, Jayewardene D, Wan B, et al. Increasing the tolerance of DCD hearts to warm ischemia by pharmacological postconditioning. Am J Transpl. 2014;14(8):1744–52. 10.1111/ajt.12782. 10.1111/ajt.12782 25040306

[9] Iyer A, Gao L, Doyle A, Rao P, Cropper JR, Soto C, et al. NormothermicEx VivoPerfusion Provides Superior Organ Preservation and enables viability Assessment of hearts from DCD donors. Am J Transplant. 2015;15(2):371–80. 10.1111/ajt.12994. 25612491 10.1111/ajt.12994

[10] Dhital KK, Iyer A, Connellan M, Chew HC, Gao L, Doyle A, et al. Adult heart transplantation with distant procurement and ex-vivo preservation of donor hearts after circulatory death: a case series. Lancet. 2015;385(9987):2585–91. 10.1016/s0140-6736(15)60038-1. 25888085 10.1016/S0140-6736(15)60038-1

[11] Messer S, Page A, Axell R, Berman M, Hernández-Sánchez J, Colah S, et al. Outcome after heart transplantation from donation after circulatory-determined death donors. J Heart Lung Transpl. 2017;36(12):1311–8. 10.1016/j.healun.2017.10.021. 10.1016/j.healun.2017.10.021 29173394

[12] Nilsson J, Jernryd V, Qin G, Paskevicius A, Metzsch C, SjöBerg T, Steen S. A nonrandomized open-label phase 2 trial of nonischemic heart preservation for human heart transplantation. Nat Commun. 2020;11(1). 10.1038/s41467-020-16782-9. 10.1038/s41467-020-16782-9 PMC7293246 32532991

[13] McGiffin DC, Kure CE, Macdonald PS, Jansz PC, Emmanuel S, Marasco SF, et al. Hypothermic oxygenated perfusion (HOPE) safely and effectively extends acceptable donor heart preservation times: results of the Australian and New Zealand trial. J Heart Lung Transplantation. 2024;43(3):485–95. 10.1016/j.healun.2023.10.020. 10.1016/j.healun.2023.10.020 37918701

[14] Louca J, Öchsner M, Shah A, Hoffman J, Vilchez FG, Garrido I, et al. The international experience of in-situ recovery of the DCD heart: a multicentre retrospective observational study. EClinicalMedicine. 2023;58:101887. 10.1016/j.eclinm.2023.101887. 36911270 10.1016/j.eclinm.2023.101887 PMC9995283

[15] Messer S, Cernic S, Page A, Berman M, Kaul P, Colah S, et al. A 5-year single-center early experience of heart transplantation from donation after circulatory-determined death donors. J Heart Lung Transplantation. 2020;39(12):1463–75. 10.1016/j.healun.2020.10.001. 10.1016/j.healun.2020.10.001 33248525

[16] Ahmed HF, Kulshrestha K, Kennedy JT, Gomez-Guzman A, Greenberg JW, Hossain MM, et al. Donation after circulatory death significantly reduces waitlist times while not changing post-heart transplant outcomes: a United Network for Organ Sharing Analysis. J Heart Lung Transpl. 2024;43(3):461–70. 10.1016/j.healun.2023.10.013. 10.1016/j.healun.2023.10.013 PMC10922468 37863451

[17] Hess NR, Hong Y, Yoon P, Bonatti J, Sultan I, Serna-Gallegos D, et al. Donation after circulatory death improves probability of heart transplantation in waitlisted candidates and results in post-transplant outcomes similar to those achieved with brain-dead donors. J Thorac Cardiovasc Surg. 2024;167(5):1845–e6012. 10.1016/j.jtcvs.2023.09.012. 37714368 10.1016/j.jtcvs.2023.09.012 PMC11411462

[18] Joshi Y, MacLean C, Emmanuel S, Wang K, Soto C, Villanueva J et al. Australian outcomes from heart transplantation in the machine perfusion era. Annals Cardiothorac Surg. 2024. doi: 10.21037/acs-2024-dcd-0074 10.21037/acs-2024-dcd-0074 PMC11618123 39649629

[19] Chew HC, Iyer A, Connellan M, Scheuer S, Villanueva J, Gao L, et al. Outcomes of Donation after Circulatory Death Heart transplantation in Australia. J Am Coll Cardiol. 2019;73(12):1447–59. 10.1016/j.jacc.2018.12.067. 30922476 10.1016/j.jacc.2018.12.067

[20] Joshi Y, Fritis-Lamora R, Wang K, Villanueva J, Kim S, Kasavaraj A, et al. Donation after Circulatory Death Heart Transplantation - Withdrawal practices and Location Matter! J Heart Lung Transplantation. 2024;43:S123. 10.1016/j.healun.2024.02.248.

[21] Ran G, Wall AE, Narang N, Khush KK, Hoffman JRH, Zhang KC, Parker WF. Post-transplant survival after normothermic regional perfusion versus direct procurement and perfusion in donation after circulatory determination of death in heart transplantation. J Heart Lung Transplantation. 2024;43(6):954–62. 10.1016/j.healun.2024.02.1456. 10.1016/j.healun.2024.02.1456 PMC11090717 38423416

[22] Bakhtiyar SS, Sakowitz S, Mallick S, Curry J, Benharash P. Heart Transplantation after Donation after Circulatory Death: early United States experience. Ann Thorac Surg. 2024;118(2):484–93. 10.1016/j.athoracsur.2024.05.013. 38815848 10.1016/j.athoracsur.2024.05.013

[23] Siddiqi HK, Trahanas J, Xu M, Wells Q, Farber-Eger E, Pasrija C, et al. Outcomes of Heart Transplant Donation after Circulatory Death. J Am Coll Cardiol. 2023;82(15):1512–20. 10.1016/j.jacc.2023.08.006. 37793748 10.1016/j.jacc.2023.08.006

[24] Krishnan A, Ruaengsri C, Guenthart BA, Shudo Y, Wang H, Ma MR, et al. Beating heart transplant procedures using organs from Donors with Circulatory Death. JAMA Netw Open. 2024;7(3):e241828. 10.1001/jamanetworkopen.2024.1828. 38466306 10.1001/jamanetworkopen.2024.1828 PMC10928498

[25] Alomari M, Garg P, Yazji JH, Wadiwala IJ, Alamouti-Fard E, Hussain MWA, et al. Is the Organ Care System (OCS) still the First Choice with Emerging New Strategies for Donation after Circulatory Death (DCD) in heart transplant? Cureus. 2022. 10.7759/cureus.26281. 35754437 10.7759/cureus.26281 PMC9229932

[26] Krishnan A, Kasinpila P, Wang H, Ruaengsri C, Shudo Y, Jackson E, Woo YJ. First-in-human beating-heart transplant. JTCVS Techniques. 2023;19:80–5. 10.1016/j.xjtc.2023.02.015. 37324334 10.1016/j.xjtc.2023.02.015 PMC10267812

[27] Landymore RW, Bayes AJ, Murphy JT, Fris JH. Preconditioning prevents myocardial stunning after cardiac transplantation. Ann Thorac Surg. 1998;66(6):1953–7. 10.1016/S0003-4975(98)00902-3. 9930475 10.1016/s0003-4975(98)00902-3

[28] Robertson JA. The dead donor rule. Hastings Cent Rep. 1999;29(6):6–14. 10641238

[29] Truog RD, Robinson WM. Role of brain death and the dead-donor rule in the ethics of organ transplantation. Crit Care Med. 2003;31(9):2391–6. 10.1097/01.Ccm.0000090869.19410.3c. 14501972 10.1097/01.CCM.0000090869.19410.3C

[30] James L, Parent B, Moazami N, Smith DE. COUNTERPOINT: does Normothermic Regional Perfusion violate the ethical principles underlying organ procurement? No. Chest. 2022;162(2):290–2. 10.1016/j.chest.2022.03.011. 35940652 10.1016/j.chest.2022.03.011

[31] DeCamp M, Snyder Sulmasy L, Fins JJ. POINT: does Normothermic Regional Perfusion violate the ethical principles underlying organ procurement? Yes. Chest. 2022;162(2):288–90. 10.1016/j.chest.2022.03.012. 35940651 10.1016/j.chest.2022.03.012

[32] Bekki Y, Croome KP, Myers B, Sasaki K, Tomiyama K. Normothermic Regional Perfusion can improve both utilization and outcomes in DCD Liver, kidney, and pancreas transplantation. Transpl Direct. 2023;9(3):e1450. 10.1097/txd.0000000000001450. 10.1097/TXD.0000000000001450 PMC9945290 36845854

[33] Watson CJE, Hunt F, Messer S, Currie I, Large S, Sutherland A, et al. In situ normothermic perfusion of livers in controlled circulatory death donation may prevent ischemic cholangiopathy and improve graft survival. Am J Transpl. 2019;19(6):1745–58. 10.1111/ajt.15241. 10.1111/ajt.15241 30589499

[34] Thomas J, Chen Q, Roach A, Wolfe S, Osho AA, Sundaram V, et al. Donation after circulatory death heart procurement strategy impacts utilization and outcomes of concurrently procured abdominal organs. J Heart Lung Transplantation. 2023;42(7):993–1001. 10.1016/j.healun.2023.02.1497. 10.1016/j.healun.2023.02.1497 PMC11181754 37037750

[35] Cain MT, Park SY, Schäfer M, Hay-Arthur E, Justison GA, Zhan QP, et al. Lung recovery utilizing thoracoabdominal normothermic regional perfusion during donation after circulatory death: the Colorado experience. JTCVS Tech. 2023;22:350–8. 10.1016/j.xjtc.2023.09.027. 38152164 10.1016/j.xjtc.2023.09.027 PMC10750961

[36] Malas J, Chen Q, Thomas J, Emerson D, Megna D, Esmailian F, et al. The impact of thoracoabdominal normothermic regional perfusion on early outcomes in donation after circulatory death lung transplantation. J Heart Lung Transpl. 2023;42(8):1040–4. 10.1016/j.healun.2023.04.009. 10.1016/j.healun.2023.04.009 PMC10524220 37098376

[37] Omelianchuk A, Capron AM, Ross LF, Derse AR, Bernat JL, Magnus D. Neither ethical nor prudent: why not to choose Normothermic Regional Perfusion. Hastings Cent Rep. 2024;54(4):14–23. 10.1002/hast.1584. 38768312 10.1002/hast.1584

[38] Wall AE, Fiedler A, Karp S, Shah A, Testa G. Applying the ethical framework for donation after circulatory death to thoracic normothermic regional perfusion procedures. Am J Transpl. 2022;22(5):1311–5. 10.1111/ajt.16959. 10.1111/ajt.16959 35040263

[39] Royo-Villanova M, Miñambres E, Sánchez JM, Torres E, Manso C, Ballesteros MÁ, et al. Maintaining the permanence principle of death during normothermic regional perfusion in controlled donation after the circulatory determination of death: results of a prospective clinical study. Am J Transplant. 2024;24(2):213–21. 10.1016/j.ajt.2023.09.008. 37739346 10.1016/j.ajt.2023.09.008

[40] Frontera JA, Lewis A, James L, Melmed K, Parent B, Raz E, et al. Thoracoabdominal normothermic regional perfusion in donation after circulatory death does not restore brain blood flow. J Heart Lung Transpl. 2023. 10.1016/j.healun.2023.05.010. 10.1016/j.healun.2023.05.010 37211334

[41] Louca JO, Manara A, Messer S, Öchsner M, McGiffin D, Austin I, et al. Getting out of the box: the future of the UK donation after circulatory determination of death heart programme. EClinicalMedicine. 2023;66:102320. 10.1016/j.eclinm.2023.102320. 38024476 10.1016/j.eclinm.2023.102320 PMC10679474

[42] Steen S, Paskevicius A, Liao Q, Sjöberg T. Safe orthotopic transplantation of hearts harvested 24 hours after brain death and preserved for 24 hours. Scandinavian Cardiovasc J. 2016;50(3):193–200. 10.3109/14017431.2016.1154598. 10.3109/14017431.2016.1154598 PMC4898163 26882241

[43] Rega F, Lebreton G, Para M, Michel S, Schramm R, Begot E, et al. Hypothermic oxygenated perfusion of the donor heart in heart transplantation: the short-term outcome from a randomised, controlled, open-label, multicentre clinical trial. Lancet. 2024;404(10453):670–82. 10.1016/S0140-6736(24)01078-X. 39153817 10.1016/S0140-6736(24)01078-X

[44] Moeslund N, Ertugrul IA, Hu MA, Dalsgaard FF, Ilkjaer LB, Ryhammer P, et al. Ex-situ oxygenated hypothermic machine perfusion in donation after circulatory death heart transplantation following either direct procurement or in-situ normothermic regional perfusion. J Heart Lung Transpl. 2023;42(6):730–40. 10.1016/j.healun.2023.01.014. 10.1016/j.healun.2023.01.014 36918339

[45] Brouckaert J, Vandendriessche K, Degezelle K, Van de Voorde K, De Burghgraeve F, Desmet L, et al. Successful clinical transplantation of hearts donated after circulatory death using direct procurement followed by hypothermic oxygenated perfusion: a report of the first 3 cases. J Heart Lung Transplantation. 2024. 10.1016/j.healun.2024.07.018. 10.1016/j.healun.2024.07.018 39069162

[46] Nadel J, Scheuer S, Kathir K, Muller D, Jansz P, Macdonald P. Successful transplantation of high-risk cardiac allografts from DCD donors following ex vivo coronary angiography. J Heart Lung Transpl. 2020;39(12):1496–9. 10.1016/j.healun.2020.08.019. 10.1016/j.healun.2020.08.019 33051105

[47] Meredith T, Scheuer S, Hoffman M, Joshi Y, Kathir K, Gunalingam B, et al. Coronary angiography of the ex-situ beating donor heart in a portable organ care system. Catheter Cardiovasc Interv. 2022;100(7):1252–60. 10.1002/ccd.30455. 36321629 10.1002/ccd.30455 PMC10091975

[48] Radakovic D, Karimli S, Penov K, Schade I, Hamouda K, Bening C, et al. First clinical experience with the novel cold storage SherpaPak™ system for donor heart transportation. J Thorac Dis. 2020;12(12):7227–35. 10.21037/jtd-20-1827. 33447411 10.21037/jtd-20-1827 PMC7797872

[49] Moayedifar R, Shudo Y, Kawabori M, Silvestry S, Schroder J, Meyer DM, et al. Recipient outcomes with extended Criteria Donors using Advanced Heart Preservation: an analysis of the GUARDIAN-Heart Registry. J Heart Lung Transpl. 2024;43(4):673–80. 10.1016/j.healun.2023.12.013. 10.1016/j.healun.2023.12.013 38163452

